# Supplementary material for: A surge of p-values between 0.041 and 0.049 in recent decades (but negative results are increasing rapidly too)
Source: PeerJ. 2015 Jan 22;3:e733. doi: 10.7717/peerj.733 (PMC4312079; doi:10.7717/peerj.733)
Supplement: Supplemental Information [file peerj-03-733-s007.zip › Supplementary material/Supplementary_Figures.pdf]

Supplementary figures for the paper
A surge of *p*-values between 0.041 and 0.049 in recent decades (but negative results are increasing rapidly too)

JCF de Winter and D Dodou

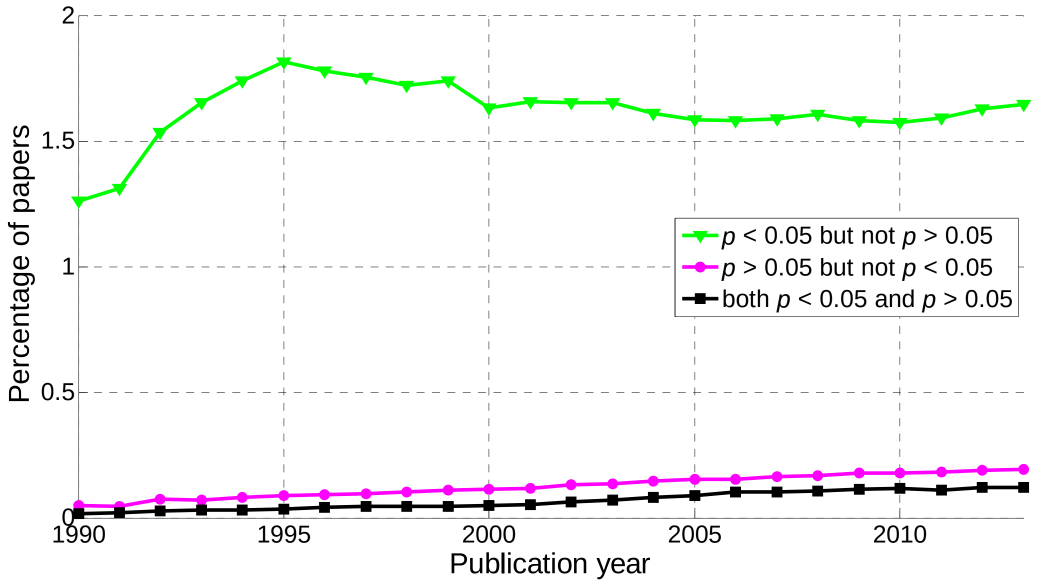

Figure S1. Percentage of papers reporting $p < 0.05$ and not $p > 0.05$, percentage of papers reporting $p < 0.05$ and not $p > 0.05$, and percentage of papers reporting both $p < 0.05$ and $p > 0.05$ per publication year.

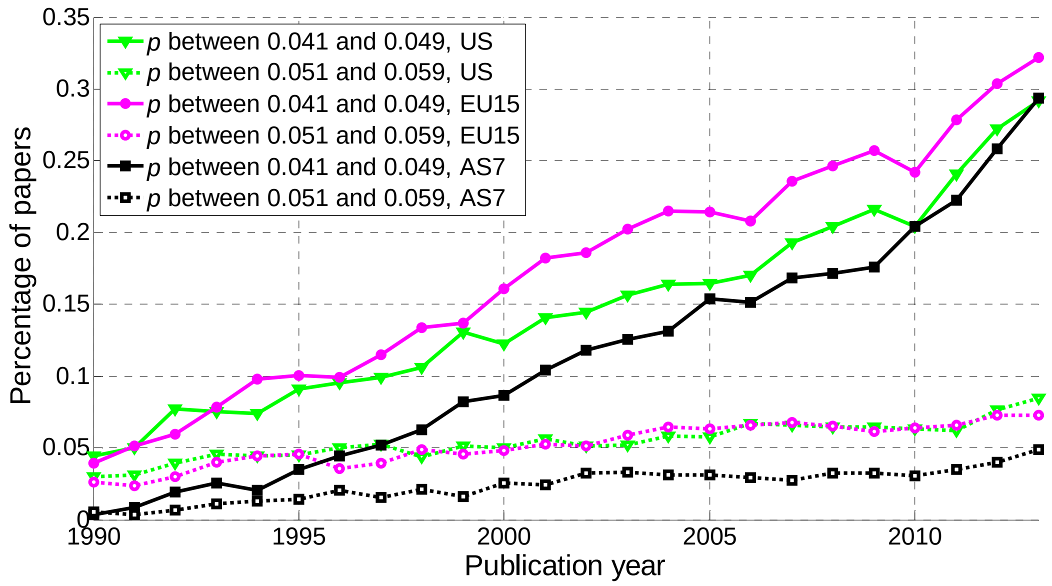

Figure S2. Percentage of papers reporting a *p*-value between 0.041 and 0.049 and percentage of papers reporting a *p*-value between 0.051 and 0.059 per publication year, for three world regions.

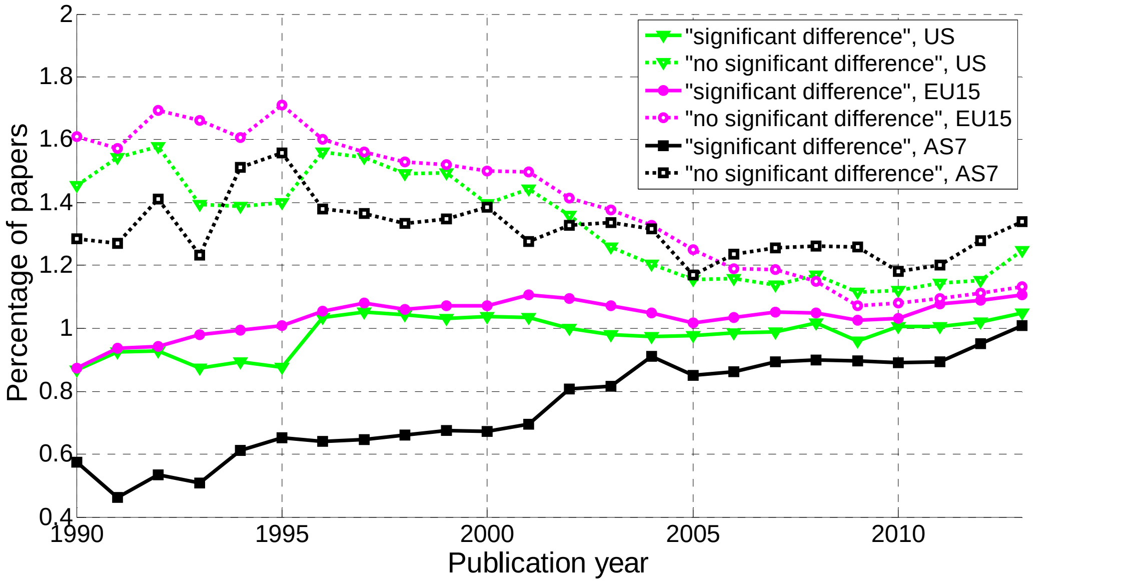

Figure S3. Percentage of papers reporting "significant difference" and percentage of papers reporting "no significant difference" per publication year, for three world regions.

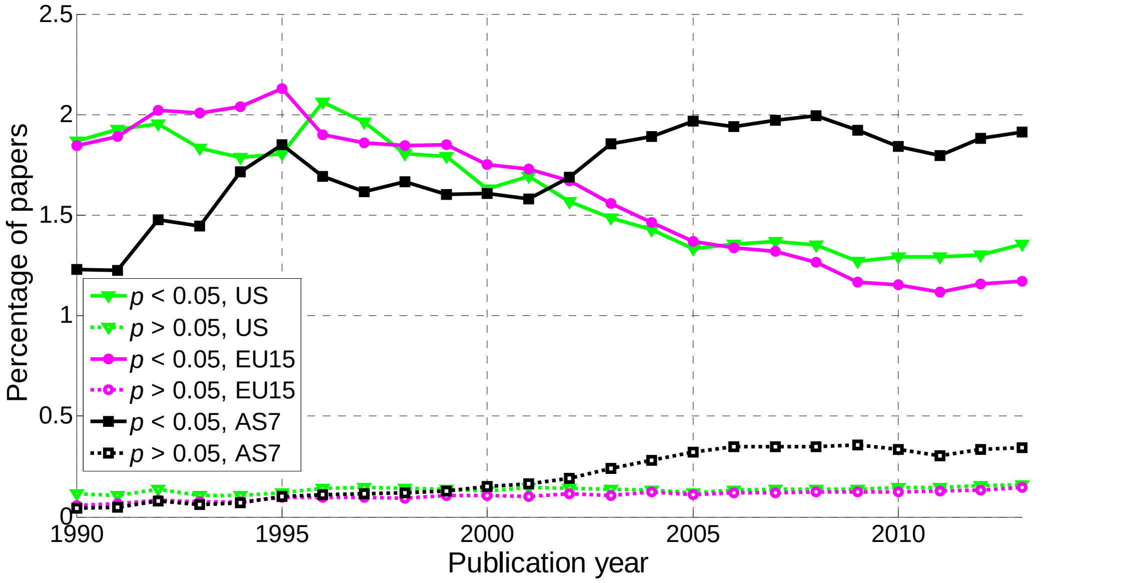

Figure S4. Percentage of papers reporting $p < 0.05$ and percentage of papers reporting $p > 0.05$ per publication year, for three world regions.

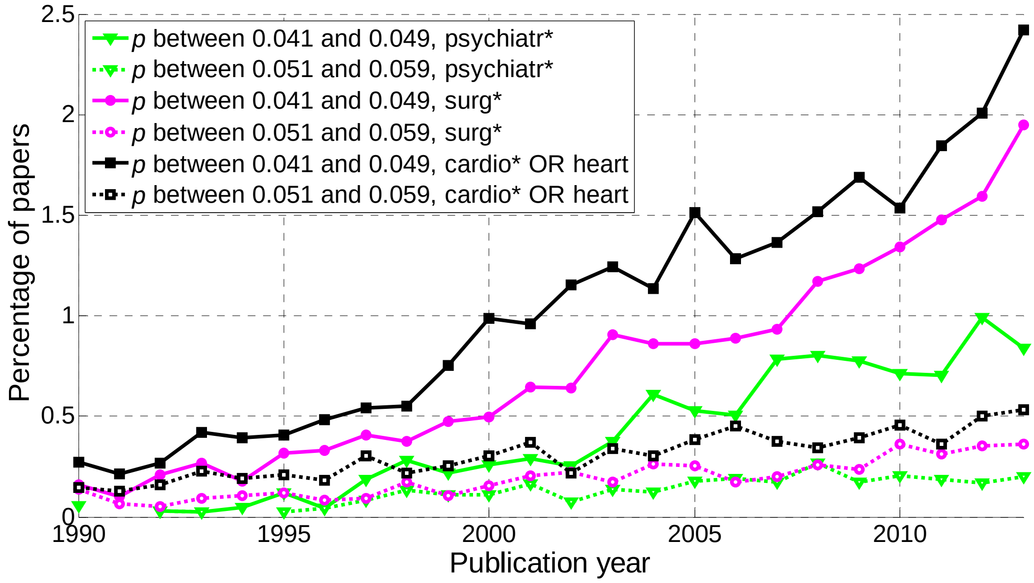

Figure S5. Percentage of papers reporting a *p*-value between 0.041 and 0.049 and percentage of papers reporting a *p*-value between 0.051 and 0.059 per publication year, for three indicative subfields of biological sciences.

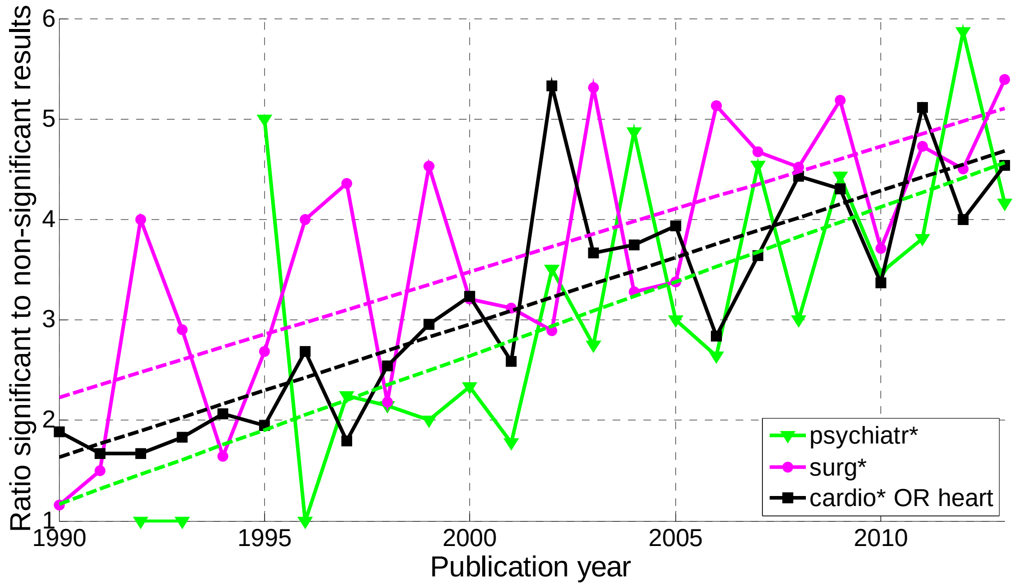

Figure S6. Ratio of *p*-values between 0.041 and 0.049 to *p*-values between 0.051 and 0.059 per publication year, for three indicative subfields of biological sciences. The dashed lines represent the results of a linear regression analysis.
